# Beyond PTSD: clinical decision-making in trauma-related disorders

**DOI:** 10.1192/j.eurpsy.2026.10269

**Published:** 2026-07-31

**Authors:** I. Riboldi

**Affiliations:** 1 Medicine and Surgery, University of Milano-Bicocca; 2 Psychiatry, Fondazione IRCCS San Gerardo dei Tintori, Monza, Italy

## Abstract

**Background:**

Trauma-related disorders are common in psychiatric practice and often present with heterogeneous symptom profiles, high comorbidity, and significant functional impairment. Beyond canonical post-traumatic stress presentations, complex, dissociative, and somatic manifestations are increasingly encountered, frequently shaped by developmental trauma, repeated interpersonal exposure, and ongoing psychosocial stressors. These features may complicate recognition, risk assessment, and treatment planning across diverse clinical settings.

**Aims:**

A pragmatic, clinically oriented framework is presented for (i) identifying trauma-related disorders and relevant specifiers, (ii) differentiating them from overlapping conditions (e.g., mood, anxiety, psychotic, personality, and functional disorders), and (iii) implementing stepped, evidence-based interventions tailored to symptom clusters, comorbidity, and patient preferences.

**Approach:**

Current diagnostic frameworks and guideline-concordant treatment evidence are synthesized, and clinical decision points applicable to routine care are integrated. Particular attention is given to structured assessment of trauma exposure and symptom profiles, dissociation, sleep, and substance use, alongside formulation-based case conceptualization and risk management for self-harm, suicidality, and re-traumatization. Emphasis is placed on transdiagnostic mechanisms, including threat processing, avoidance, and emotion dysregulation, and on trauma-informed communication to enhance engagement and retention.

**Clinical Implications:**

A phased, personalized care pathway is proposed. Initial stabilization focuses on safety, psychoeducation, sleep and arousal regulation, and management of comorbid depression, anxiety, and substance use. Readiness for trauma-focused therapy is addressed through selection and sequencing of first-line psychotherapies, including trauma-focused CBT, EMDR, prolonged exposure, and cognitive processing therapy, with adaptations for dissociation, complex presentations, and ongoing stress. Pharmacological strategies are discussed as adjuncts targeting specific symptoms such as hyperarousal, nightmares, and comorbid mood and anxiety disorders, with caution regarding polypharmacy and agents with dependence potential. Special considerations are outlined for developmental trauma, perinatal contexts, migration-related trauma, and culturally sensitive assessment.

**Conclusions:**

A structured yet flexible clinical approach is required for trauma-related disorders, balancing diagnostic precision with formulation, prioritizing safety and engagement, and applying stepped, evidence-based interventions. Trauma-informed, mechanism-focused pathways may improve outcomes, reduce chronicity, and support integrated care across psychiatric services.

**Disclosure of Interest:**

None Declared

